# Stochastic dynamics of social patch foraging decisions

**DOI:** 10.1103/physrevresearch.4.033128

**Published:** 2022-08-15

**Authors:** Subekshya Bidari, Ahmed El Hady, Jacob D. Davidson, Zachary P. Kilpatrick

**Affiliations:** Department of Applied Mathematics, University of Colorado Boulder, Colorado 80309, USA; Princeton Neuroscience Institute, Princeton, New Jersey 08540, USA;; Department of Collective Behavior, Max Planck Institute for Animal Behavior, Konstanz D-78457, Germany;; Cluster for Advanced Study of Collective Behavior, Max Planck Institute for Animal Behavior, Konstanz D-78457, Germany; Department of Collective Behavior, Max Planck Institute for Animal Behavior, Konstanz D-78457, Germany; Department of Applied Mathematics, University of Colorado, Boulder, Colorado 80309, USA

## Abstract

Animals typically forage in groups. Social foraging can help animals avoid predation and decrease their uncertainty about the richness of food resources. Despite this, theoretical mechanistic models of patch foraging have overwhelmingly focused on the behavior of single foragers. In this study, we develop a mechanistic model that accounts for the behavior of individuals foraging together and departing food patches following an evidence accumulation process. Each individual’s belief about patch quality is represented by a stochastically accumulating variable, which is coupled to another’s belief to represent the transfer of information. We consider a cohesive group, and model information sharing by considering both intermittent pulsatile coupling (only communicate decision to leave) and continuous diffusive coupling (communicate throughout the deliberation process). Groups employing pulsatile coupling can obtain higher foraging efficiency, which depends more strongly on the coupling parameter compared to those using diffusive coupling. Conversely, groups using diffusive coupling are more robust to changes and heterogeneities in belief weighting and departure criteria. Efficiency is measured by a reward rate function that balances the amount of energy accumulated against the time spent in a patch, computed by solving an ordered first passage time problem for the patch departures of each individual. Using synthetic departure time data, we can distinguish between the two modes of communication and identify the model parameters. Our model establishes a social patch foraging framework to identify deliberative decision strategies and forms of social communication, and to allow model fitting to field data from foraging animal groups.

## I. INTRODUCTION

Foraging is a ubiquitous behavior performed by all animals for survival, and many species forage in groups. Communication among group members is essential to maintain cohesion and share important information, e.g, available resources or possible threats [1,2]. Group foraging can offer increased vigilance and protection against predators [3–5], more rapid reduction of uncertainty about the density or quality of resources [6,7], and enhanced ability to capture prey [8]. Since resource distributions can be spatially heterogeneous [9–11], a common modeling assumption takes resources distributed in patches [12–14] with low availability between. Animals exploit resources in a patch until departing for another patch. A key question is how animals determine if and when to depart a patch.

Many animals forage in groups and use social information to shape their movement and resource exploitation decisions (e.g., baboons and capuchins [15,16]), but mechanistic models of patch departure deliberation have mainly focused on individual foragers [17,18]. Here we introduce a model of social patch foraging and departure deliberation, describing how the social information modulates the process of two or more foragers deciding when to leave a patch. Social foraging differs from individual foraging because it alters both resource availability and a forager’s behavior [19]. The proximity of conspecifics may alter foraging performance through information sharing [20–22]; scrounging [23,24]; the opportunity for kleptoparasitism [25,26]; or attention required for predator detection (e.g., the collective detection hypothesis [27–29]). Individuals’ response to their social companions recasts the relationship between their foraging performance and the group density, potentially deviating from the typical “ideal free distribution” model [30]. Group foragers often simultaneously share patches [24,31–35], which can increase or decrease food uptake rate, depending on information sharing or social learning [36]. Such arrangements are especially beneficial to individuals who exploit their conspecifics’ discoveries for their own gains. For example, high-ranking birds in dominance hierarchies can improve their average food intake rate in this fashion [37], while lower-ranked birds’ average uptake rate is reduced compared to the rest of the group. Nevertheless, group foraging can decrease the likelihood of any bird going without food. Animals may also change their level of group cohesion depending on resource availability. Finches tend to share patches when food is scarce (when the probability of starvation was high) but search individually when food is plentiful [34]. Thus, there is a rich landscape of behaviors connecting social information sharing and energy yields from patch foraging, which we quantitatively formalize here.

Bayesian models have proposed optimal ways to combine individual and social information in order to gain information about the environment [38]. Theory has been used to show how group structure and communication affect behavior, determining when differences in information drive a group to split apart, or the fraction of informed individuals needed to lead a group to a known location [39,40]. Other studies have used models of contagion [41,42] to examine how a behavior spreads through a group [43]. One observed advantage of information sharing in groups is that multiple estimates of the same quantity (e.g., chemical gradients or food density) reduce uncertainty arising from measurement or internal noise [44,45]. While the efficiency of patch foraging decisions can be limited by uncertainty and stochasticity [18], such effects may be ameliorated by social communication. To account for uncertainty and noise in decision processes, one can treat foraging decisions as the result of an evidence accumulation process as in [17]. The stochastic departure time of each individual can be calculated from an accumulation to bound process, wherein a drifting and diffusing variable represents a current belief and a fixed bound triggers a patch departure [17]. Such drift-diffusion models have been successful in untangling the strategies animals use to make binary perceptual choices [46]. Decision times can then be obtained as the solutions to first passage time problems using methods from stochastic processes and asymptotics [47–49]. Behaviors described by stochastic bound-crossing problems commonly emerge in decision making as well as neural spiking [50], search processes [51], and biomolecular trafficking [52]. In our study, we analyze bound crossing for coupled diffusiontype processes in the case of different types of coupling. Work in this direction could shed light on collective processes that involve information exchange between stochasticallyevolving system components.

Leveraging the drift-diffusion modeling framework, we formulate a social patch foraging model and study how information sharing in animal groups and heterogeneity of beliefs affect patch-leaving decisions and the efficiency of group decisions. In the model, we consider a cohesive foraging group and two different ways individuals might share their evolving beliefs—diffusive or pulsatile coupling. Diffusive coupling represents continuous sharing of information described previously as an “ideal group” for human group decision making [44,53]) and migrating animals collectives [54,55]. Pulsatile coupling shares information only when a decision is made [56,57]. Strengthening coupling between individuals generally improves the efficiency of group decisions (i.e., increasing the average rate of energy intake) in either model by coordinating individual departure decisions. The diffusively coupled model is generally more robust to suboptimal, detuned parameter choices. Precise alignment of patch departure time is more important for the efficiency of cohesive foraging groups than the tuning individual departure times. When individual decision times differ, agents wait for one another after they stop foraging in the patch, and this waiting time reduces the overall time spent foraging. We also develop model-fitting methods and determine identifiability using synthetic data (i.e., patch departure times generated by the models), showing the more sensitive pulsatile coupled model is more difficult to identify from data.

Our paper considers combining deliberative evidence-accumulation processes with information sharing in the context of patch-leaving decisions. By specifying different forms of information sharing, we have identified how the nuances of social communication shape patch-departure coordination within groups. Not only are these models relevant to patch departure but also other stay/go decisions like predator escape, mating initiation, and habitat search. Finally, our model-fitting framework can accurately constrain animals’ deliberation strategies from experimental field data and also infer modes of communications they use during natural social patch foraging decisions.

## II. MODEL AND METHODS

**FIG. 1.**
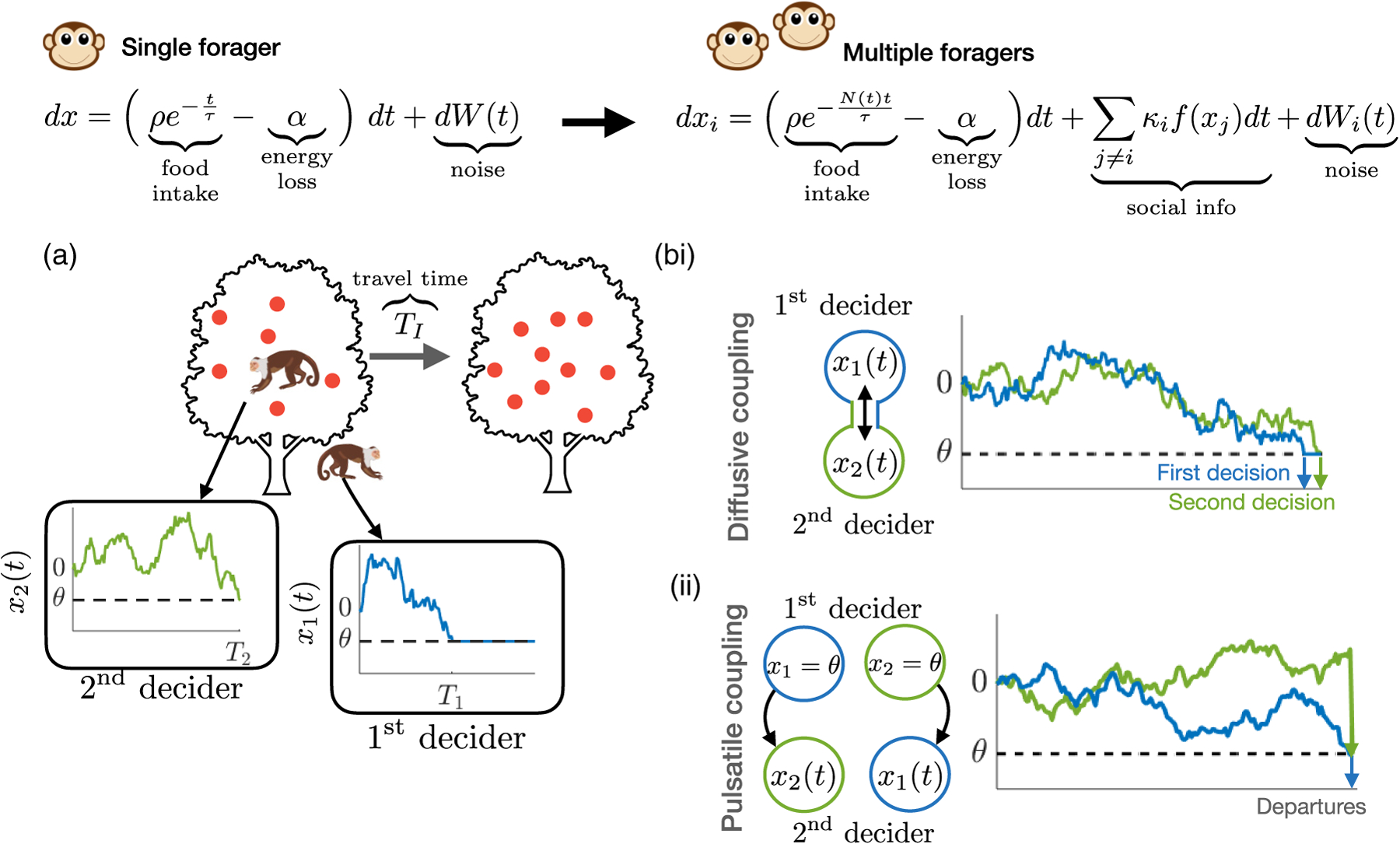
Evidence accumulation model for patch leaving in individuals and groups. (a) Schematic of patch departure decision in a group with two agents. (b) (i) *Diffusive coupling*—agents communicate their beliefs throughout the evidence accumulation process to their neighbors. (ii) *Pulsatile coupling*—agents only communicate their decision to leave the patch to their neighbors.

Our stochastic model describes *N* agents’ beliefs about the quality of the patch where they are currently foraging (Fig. 1). We consider a cohesive group where all individuals in the group leave the patch together once all group members have made a departure decision. Thus, there are two key time points for any individual: when that individual *i* decides to leave and stops foraging (*T*_*i*_), and when the whole group leaves (*T*_*N*_). The group leaving time is the maximum individual decision time among the *N* group members [*T*_*N*_ = max({*T*_*i*_})], and therefore a high variance in *T*_*i*_ leads to a low average returns, due to the time early individuals spend waiting for others to depart. Consider the concrete example of capuchin monkeys foraging on fruit trees. The time *T*_*i*_ is when individual *i* comes down from the tree, and the time *T*_*N*_ is when all individuals have come down from the tree and the group leaves together for another tree.

Prior to introducing models with information coupling, we discuss our model of a single forager (*N* = 1). In previous paper, we have shown that the decision of whether or not to depart a patch containing food can be described using Bayesian evidence accumulation [18]. Stochastic variables representing probabilities associated with different possible food densities evolve in response to the time spent by the forager, its food encounters, and food depletion. This generally evolves as a drift-jump process, but when food encounters are rapid, a diffusion approximation results in a drift-diffusion process representing the stochasticity of food encounters and any additional sensory/memory noise associated with an agent’s imperfect estimate of food availability [17]. Rather than surveying the detailed suite of individual forager models we derived in previous paper, we opt for a simple and approximate version of this evidence accumulation process. See the Appendix for details on deriving the drift term as the first moment of the depletion process arising from resource consumption. Also, while a diffusion approximation of the ideal Bayesian model would have noise amplitude that changes over time in a specific way, to identify models of evidence accumulation from data, it is necessary to make this amplitude parameterizable and constant. The belief *x* of a foraging agent is driven by the noisy sampling of a depleting resource in a patch, which starts at density *ρ* and decays with timescale *τ* as the agent forages. Our previous paper considered an optimal Bayesian description of the belief of patch food density [18]. This motivates the use of this more general form of model in which the decision variable *x* represents not patch quality belief but rather an animal’s motivation to stay in the patch, driven food encounters, food decrementing, and internal noise [17]. Negative values of *x* imply that the patch is no longer profitable and decreases in *x* eventually lead to the agent reaching threshold and leaving the patch. Note that the drift in our model represents the average rate of resource consumption; due to the stochastic nature of foraging, the agent will not encounter food with perfect regularity, and the agent may not maintain a perfect estimate of available food. With this, the evidence accumulation process of a single forager evolves according to the following stochastic differential equation (SDE) with initial and stopping conditions,

(1) $$dx=\left(\rho e^{-\frac{t}{\tau }}-\alpha \right)\;dt+\sqrt{2B}\;dW(t),$$

with *x*(0) = 0 and *x*(*T*) = *θ*, where *α* is the cost associated with foraging, and *W*(*t*) is the standard Wiener process (zero mean, variance unity). The scaling *B* captures the stochasticity in food encounters as well as the imperfect food availability estimate maintained by the forager. We might expect *B* to vary with time in precise correspondence between Eq. (1) and a diffusion approximation of a Bayesian observer, but we approximate it as constant since we are incorporating constant amplitude sensory noise as well. We assume *ρ* > *α*, i.e., the foraging cost is less than the initial energy gained from foraging the patch. The agent will depart the patch at a stochastically determined time *t* = *T* such that *x*(*T*) = *θ* < 0. We consider an “increment-decrement” patch leaving strategy, where food encounters initially increase the animal’s likelihood to stay in the patch but eventually deplete it, leading to a lack of encounters and departure [17]. Following Eq. (1), we use negative threshold values, such that food encounters move the agent away from the decision threshold, and the absence of food towards the threshold.

We incorporate information transfer among individuals to represent foraging social groups [Fig. 1(a)]. Information sharing can take the form of social cues and signals (inadvertent or intentional) emitted to influence the behavior of conspecifics [58–62] and is mathematically represented in our models as the coupling of decision states between individuals in a group [56]. Note, based on previous results in [57], it is likely that we should include an additional weak term based on information foragers might glean from their conspecifics indecision, but we omit this in the interest of focusing on models more identifiable from data. Thus, while we could potentially derive an ideal observer model based on Bayesian sequential updating, in this study we are interested in a flexible model with different kinds of information sharing and a free parameter describing variability in the resource estimate. We consider two different information-sharing mechanisms. (i) *Diffusive coupling*. Agents communicate their beliefs continuously throughout the evidence accumulation process, and are attracted to the relative beliefs of their neighbors according to an individual coupling strength *κ*_*j*_. A similar formulation has been considered previously for drift-diffusion models with constant drift [44]. The corresponding stochastic differential equations evolve as

(2a) $$dx_{i}=\left[\rho e^{-\frac{𝒩(t)t}{\tau }}-\alpha +\underset{j\neq i}{\sum }\kappa_{i}\left(\left(x_{j}-\theta_{j}\right)-\left(x_{i}-\theta_{i}\right)\right)\right]dt+\sqrt{2B}\;dW_{i}(t),$$

(2b) $$x_{i}(0)=0,\;x_{i}\left(T_{j}\right)=\theta_{i}.$$

The quantity (*x*_*i*_ − *θ*_*i*_) is the agent *i*’s distance-to-threshold, so agents are attracted to (repelled from) their decision threshold by the coupling term if other agents are at (not at) their decision threshold, promoting synchronization of agents’ decision variables and departure times of agents [Fig. 1(b)(i)]. Decision thresholds *θ*_*i*_ and coupling strengths *κ*_*i*_ can vary across individuals in the model. For instance, an individual may be more likely to assign higher value to their neighbors’ beliefs if following them yielded high reward rates in previous foraging bouts. Similarly, individuals might need more or less evidence to depart the patch based on their experience in a similar resource environment.

For individuals foraging in a group, the resource decay rate scales with the number of agents in a patch, which begins at *N*. We represent this with 𝒩(*t*), which is the decreasing counting function

(3) $$𝒩(t)=\{\begin{array}{ll} N & 0<t<T_{1}\\ N-1 & T_{1}<t<T_{2}\\ \vdots & \\ 1 & T_{N-1}<t<T_{N} \end{array},$$

where *T*_*j*_ is the time when the *j*th decider reaches their decision threshold [*x*_*i*_(*T*_*j*_) = *θ*_*i*_], after which their belief state remains there. This continues until the final agent has decided when *x*_last_(*T*_*N*_) = *θ*_*i*_. (ii) *Pulsatile coupling*. Agents only communicate a pulse of information to their neighbors when they decide to stop foraging. When an agent’s belief state reaches the threshold, neighboring agents receive a pulse in their belief state towards the threshold corresponding of size given by their coupling strength *κ*_*i*_ [Fig. 1(b)(ii)]. The belief states of agents evolve as

(4a) $$dx_{i}=\left[\rho e^{-\frac{𝒩(t)t}{\tau }}-\alpha -\underset{j\neq i}{\sum }\kappa_{i}\delta \left(x_{j}-\theta_{j}\right)\right]dt+\sqrt{2B}\;dW_{i}(t),$$

(4b) $$x_{i}(0)=0,\;x_{i}\left(T_{j}\right)=\theta_{i},$$

where 𝒩(*t*) is defined in Eq. (3) and *δ*(*x*) is the delta distribution. When the first agent decides to leave (at time *t* = *T*_1_), undecided agents (*x*_*j*_) receive a pulse of size *κ*_*j*_, propelling their belief towards their decision threshold. This may immediately trigger remaining agents to make a decision (*x*_*j*_(*T*_1_^+^) ⩽ *θ*_*j*_), or the agents may continue to accumulate evidence until reaching threshold *θ*_*j*_. Like many animals [16,63], individuals do not depart for another patch until all others in the group have decided to leave. Our model thus incorporates the notion of group cohesion into the travel bouts between patches.

## III. RESULTS

Collectives can improve their foraging efficiency by coordinating spatial movements and patch departures so the group remains cohesive [1]. We measure efficiency of the group’s decision according to the average reward rate for all group members, averaged across many patch bouts. Note that the density of food in a patch is given by $\rho e^{-\frac{𝒩(t)\cdot t}{\tau }}$, proportional to the rate at which food is encountered as identified in [18]. For simplicity, we assume a rescaling of resource and energy consumption so that the corresponding proportionality constant is unity. Assuming the rate of resource consumption by each of the agents equals the rate of resource decay,

$$\frac{dr}{dt}=\rho e^{-\frac{𝒩(t)\cdot t}{\tau }},$$

we can integrate and scale by the number of agents consuming food at any given time 𝒩(*t*) to obtain the the total food consumed by all of the agents before the time *T* = *T*_*N*_,

$$r_{N}(T)=\int_{0}^{T_{N}}𝒩(t)\frac{dr}{dt}dt\;=\rho \tau [\left(1-e^{-\frac{N\cdot T_{1}}{\tau }}\right)+e^{-\frac{N\cdot T_{1}}{\tau }}\left(1-e^{-\frac{(N-1)\left(T_{2}-T_{1}\right)}{\tau }}\right)\;+\cdots +e^{-\frac{N\cdot T_{1}}{\tau }}e^{-\frac{(N-1)\left(T_{2}-T_{1}\right)}{\tau }}\ldots \;\times \left(1-e^{-\frac{\left(T_{N}-T_{N-1}\right)}{\tau }}\right)].$$

Scaling by 𝒩(*t*) accounts for how many agents are consuming food at any given time, given the total food consumption of the group. Accounting for the travel time between patches *T*_*I*_ and a constant energy loss rate *α* due to movement, we define the reward rate in the patch as

$$RR=\frac{\langle r_{N}(T)\rangle -\alpha \left(T_{I}+\langle T\rangle \right)}{T_{I}+\langle T\rangle },$$

where the reward and departure time are averaged across realizations for a given strategy. Efficiency of a group foraging strategy is thus measured by the relative value of RR. We compute the expected reward rate in the patch by averaging the distributions of patch departure times determined from the first passage times of the threshold crossing processes defined by Eq. (2) and Eq. (4).

Before delving into the optimality of different coupling mechanisms, we compare limiting cases in a symmetric two agent system where agents have the same decision threshold *θ*_*j*_ = *θ* and coupling constant *κ*_*j*_ = *κ* for *j* = 1, 2 that is either absent (*κ* = 0) or infinite (*κ* → ∞).

### A. Perfectly coupled homogeneous two agents system

We start by comparing groups’ patch departure times (*T*_*N*_) and reward rates (RR) for various idealized conditions, including (1) No information coupling (NC), (2) Perfect diffusive coupling (*D*_∞_), and (3) Perfect pulsatile coupling (*P*_∞_).

With no information coupling (NC), the decision variable for each agent evolves as an independent and identically distributed (i.i.d) process

$$dx_{i}=\left(\rho e^{-\frac{𝒩(t)t}{\tau }}-\alpha \right)dt+\sqrt{2B}\;dW_{i}(t),$$

where *x*_*i*_(0) = 0 and *x*_*i*_(*T*_*j*_) = *θ*, and 𝒩(*t*) is a decreasing counting function as in Eq. (3).

In a perfectly diffusive coupled model (*D*_∞_) with the same thresholds, we can show that the strong limit of diffusive coupling averages out half the noise. Before either agent’s belief reaches threshold, the mean *x*_+_ = (*x*_1_ + *x*_2_)/2 and half-difference *x*_−_ = (*x*_1_ − *x*_2_)/2 evolve as

$$dx_{+}=\left(\rho {\text{e}}^{-2t/\tau }-\alpha \right)dt+\sqrt{B/2}\;dW_{1}+\sqrt{B/2}\;dW_{2},$$

$$dx_{-}=-\kappa x_{-}dt+\sqrt{B/2}\;dW_{1}-\sqrt{B/2}\;dW_{2},$$

for arbitrary *κ*. Using standard methods for Ornstein-Uhlenbeck processes [49], we see in this limit 〈*x*_−_〉 = 0 and $\langle x_{-}^{2}\rangle ={\text{lim}}_{\kappa \to \infty }\frac{B}{4\kappa }\left(1-{\text{e}}^{-4\kappa t}\right)=0$. Clearly, *x*_−_ ≡ 0 for *κ* → ∞, implying *x*_1_ = *x*_2_ = *x*_+_. Thus, each agent’s belief evolves identically due to the averaging of all agents’ drift and diffusion terms, reducing Eq. (2) to

(5) $$dx=\left(\rho e^{-\frac{2t}{\tau }}-\alpha \right)\;dt+\sqrt{B}\;dW(t),$$

where *x*(0) = 0 and *x*(*T*) = *θ*. The noise amplitude of this averaged equation is half that of the full model in Eq. (2) due to the noise-cancellation by diffusive coupling [49]. Since the agents’ decision times are identical, the group’s patch departure time is given by the first passage time of Eq. (5).

In a model with perfect-pulsatile coupling (*P*_∞_), the first decider always immediately triggers departure decisions from the remainder of the group. Thus, group decision time is given by the minimum first passage time across *j* = 1, 2 of

$$dx_{i}=\left(\rho e^{-\frac{2t}{\tau }}-\alpha \right)\;dt+\sqrt{2B}\;dW_{i}(t),$$

where *x*_*i*_(0) = 0 and *x*_*i*_(*T*_*j*_) = *θ*.

**FIG. 2.**
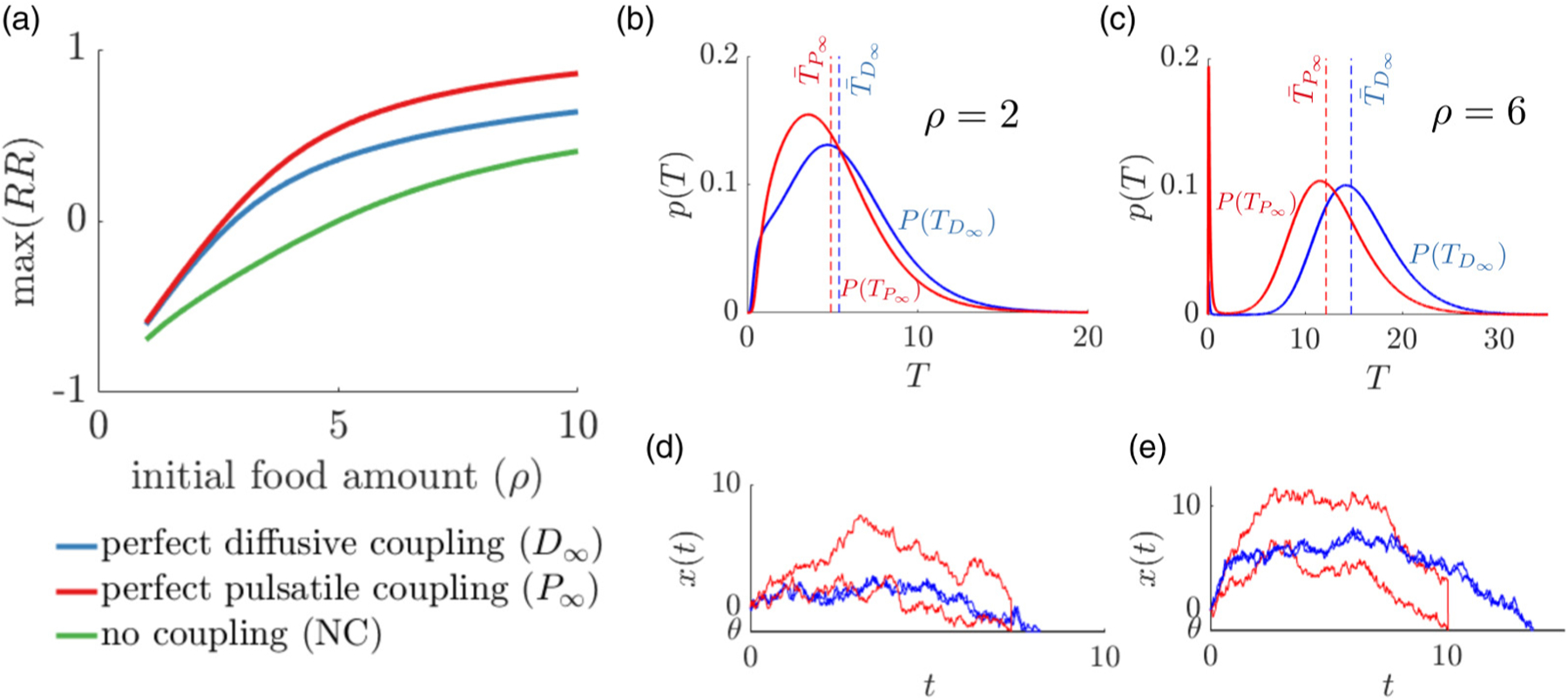
Coupling of belief states between group members increases the reward rate (*RR*). (a) Comparison of reward rate for a two-agent system: perfectly diffusively coupled (*D*_∞_), perfectly pulsatile coupled (*P*_∞_), and no information coupling (NC). For lower values of initial food, diffusive and pulsatile coupling perform similarly generating higher reward compared to the uncoupled case. When the initial food amount is higher, pulsatile coupling outperforms diffusive coupling. For fixed values of model parameters *τ* = 5, *B* = 1, *α* = 1, *T*_*I*_ = 5, and *ρ* = 2, we maximize the reward rate for the group by optimizing the patch departure threshold *θ*. [(b),(c)] Patch departure time distribution for diffusive (blue) and pulsatile (red) coupling. The dashed line shows average patch departure time. A single simulation of pulsatile (red) and diffusive coupled (blue) models when (d) *ρ* = 2 and (e) *ρ* = 6.

Compared to the no-coupling case, having information coupling increases the group reward rate [Fig. 2(a)] for optimal choices of the departure threshold *θ* of each limiting model. With infinitely strong coupling, the diffusive model has lower effective noise, but in both models, agents make simultaneous departure decisions. For high *ρ* values [Fig. 2(a)], increasing the noise amplitude increases the overall average RR, by lowering the average decision time [Figs. 2(b)–2(e)]. Note the sharp peak in the likelihood of early departure times for the pulsatile coupled model given more initial food [Fig. 2(c)]. This is because the optimal threshold *θ*^opt^ sits closer to the initial belief at *x*_*i*_(0) = 0. These results demonstrate that social coupling generally increases the average reward rate for a cohesive group, but results depend on the parameter regime. While the drift and threshold values can be tuned to maximize RR for a solitary individual or for the idealized coupling case [17], here we fix the drift, and focus on a comparison of the two coupling methods and their parameter dependence in a finite range. The departure time statistics are computed by numerically simulating the associated Fokker-Planck equations (see Appendix A2b).

### B. Decisions in diffusive coupled system

**FIG. 3.**
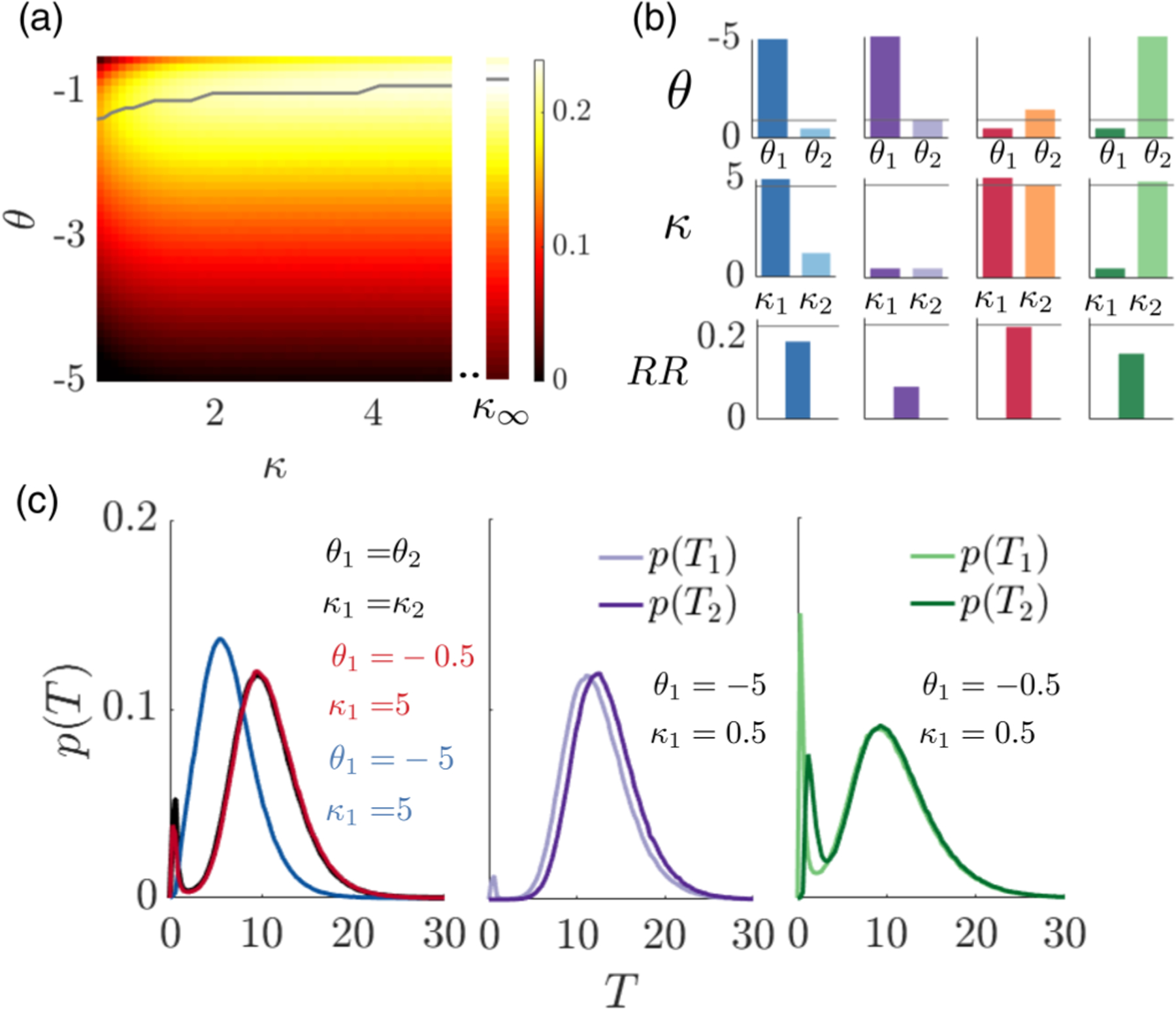
Comparison of reward rates (RRs) in a group with diffusively coupled belief states. (a) Heat map shows how reward rate varies with the decision threshold *θ* and coupling strength *κ* for a symmetric model *θ*_1_ = *θ*_2_ = *θ* and *κ*_1_ = *κ*_2_ = *κ*, and *α* = 1, *ρ* = 4, *τ* = 5, and *T*_*I*_ = 5. Decision and departure time statistics are obtained by running Monte Carlo simulations for Eq. (2) (see Appendix A2a). Grey line: Optimal threshold value *θ*^opt^ for fixed coupling strength. The line is smoothed by computing averages over a sliding window of length 3. Right column: RR for the infinite coupling limit (*κ* → ∞). (b) Asymmetric strategies. Optimal threshold *θ*_2_ (1st row), coupling *κ*_2_ (2nd row), and RR (3rd row) when fixing agent 1 threshold *θ*_1_ and coupling strength *κ*_1_. Grey line: Optimal symmetric *θ* and *κ*. (c) Patch departure decision time distributions for each group in (b).

Relaxing to finite coupling strengths, we now examine how group strategy parametrization shapes foraging efficiency. In a homogeneous group (individuals all use the same decision threshold *θ* and coupling strength *κ*) with diffusive coupling, increasing the coupling strength increases the RR, as long as the decision threshold is properly tuned (along blue line) due to noise cancellation [Fig. 3(a)].

If we break the symmetry of the diffusive coupling model (differing coupling strengths or decision thresholds), individuals may value their neighbor’s beliefs less (lower *κ*_*i*_) or may need less evidence to decide to depart the patch (*θ*_*i*_ closer to zero), which could occur due to varying experience or access to information across the group. To identify how asymmetric strategies shape group performance, we consider two agents with different decision thresholds *θ*_1_ ≠ *θ*_2_ and coupling strengths *κ*_1_ ≠ *κ*_2_. We compare four representative cases, different combinations of high and low decision thresholds (*θ*_1_) and coupling parameters (*κ*_1_) for agent 1, in which we optimize the other agent’s (*θ*_2_, *κ*_2_). When agent 1’s fixed decision threshold is mistuned but diffusive coupling is strong, the group RR can still be near optimal if agent 2 chooses a decision threshold that counteracts agent 1’s threshold being too large/small [Fig. 3(b)]. However, when agent 1’s coupling is weak, the optimal group RR is substantially suboptimal even when agent 2 compensates for decision threshold mistuning. As with symmetric groups, strong social communication is important for efficient group-level foraging. Strong coupling). pushes (pulls) the belief of the first agent with a far (close) decision threshold towards their decision threshold such that the foraging time for both agents is similar [Fig. 3(c), blue/red curves]. When agent 1 is weakly coupled to agent 2, the best strategy for the group is for agent 2 to compensate for late/hasty decisions by speeding/slowing their own, but the agents decide at different times and the group’s RRs are decreased [Fig. 3(c), purple/green curves].

### C. Decisions in pulsatile coupled system

**FIG. 4.**
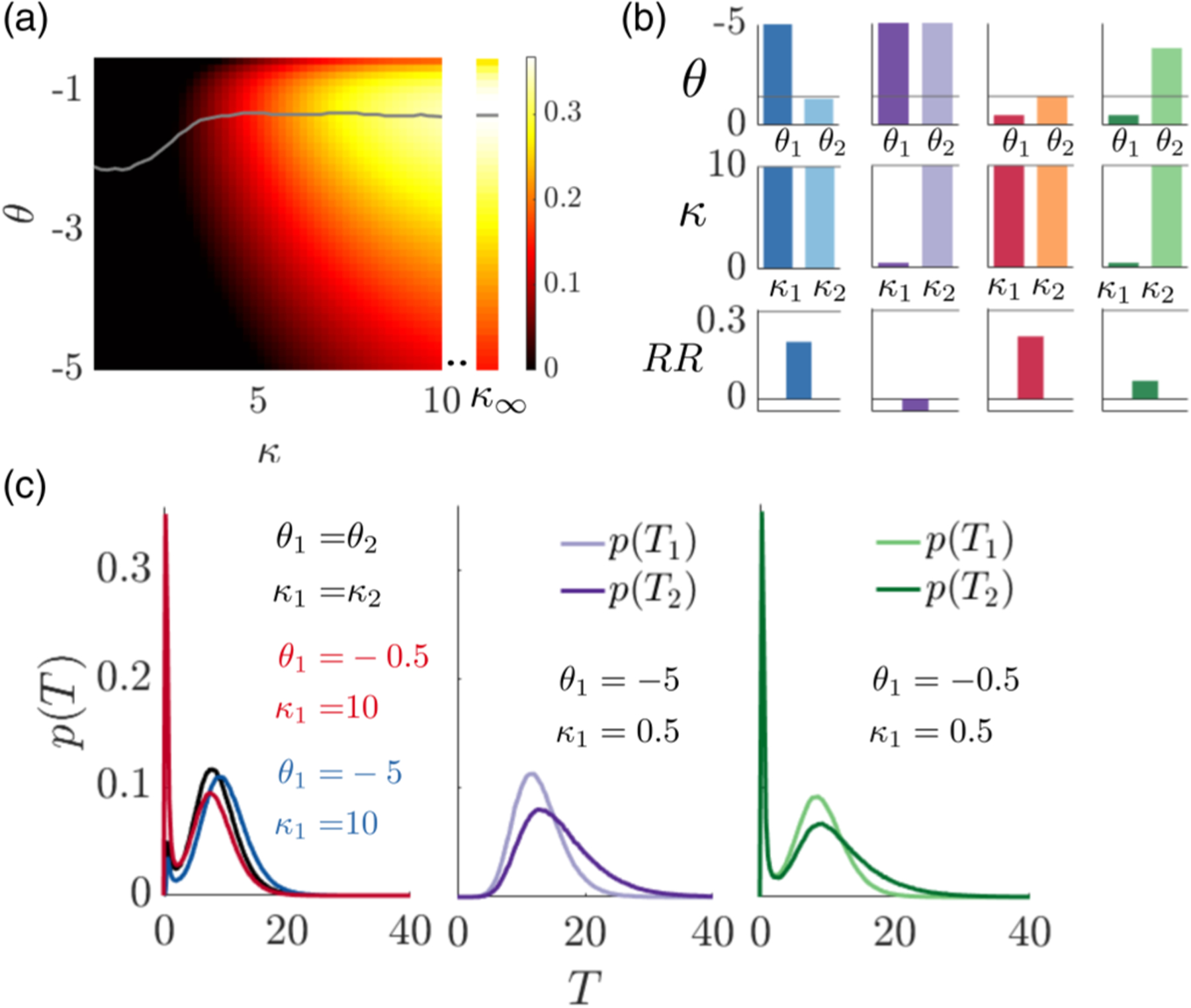
Comparison of RRs in group with pulsatile coupling. (a) RR heat map for a two agent group with the same decision threshold *θ* and coupling strength *κ*, and *α* = 1, *ρ* = 4, *τ* = 5, and *T*_*I*_ = 5. Decision and departure time statistics are obtained by running Monte Carlo simulations for Eq. (4) (see Appendix A2a). Grey line: Optimal decision threshold *θ* for a given coupling strength. The line is smoothed by computing average over a sliding window of length 3. Right column: RR in the infinite coupling limit (*κ* → ∞). (b) Asymmetric strategies. Optimal threshold *θ*_2_ (1st row), coupling *κ*_2_ (2nd row), and RR (3rd row) for fixed agent 1 threshold *θ*_1_ and coupling strength *κ*_1_. Grey line: Optimal symmetric *θ* and *κ*. (c) Patch departure decision time distributions for each group in (b).

Pulsatile coupling involves less overall information exchange and yet has a stronger dependence on the coupling parameters *κ*_*j*_ compared to diffusive coupling [Fig. 4(a)]. As with the diffusively coupled group, strong coupling increases groups’ RR in a pulsatile coupled system [Fig. 4(a)]. Moreover, the RR falls off sharply for weak coupling [compare Fig. 3(a) and Fig. 4(a)]. For asymmetric strategies, this trend is even apparent when agent 1’s coupling is weak but agent 2 employs strong compensatory coupling [Fig. 4(b), purple/green bars]. Even with strong bidirectional pulsatile coupling, heterogeneous groups attain lower RRs than heterogeneous diffusively coupled groups [compare Fig. 3(b), blue/red and Fig. 4(b), blue/red]. This indicates that while pulsatile coupling strategies can attain high RRs in the symmetric case with intermediate values of *κ*, their performance is sensitive to mistuning. Since agents only communicate their decisions to depart, one cannot compensate for the mistiming of their neighbor’s departure decisions, but can only synchronize with poor departure decision times to raise the group RR [Fig. 4(c)].

### D. Model identification and fitting

To test model identifiability and illustrate model fitting, we developed a method for inferring model parameters from synthetic data (i.e., generated by the model itself) for a group with two agents. Parameters were selected using the Bayesian maximum *a posteriori* (MAP) method [64]. Using Bayes rule and the independence of each departure time observation pair **T**^*k*^, we can write down the posterior distribution for the probability of model parameters Θ given *K* observed departure decision time pairs **T**^1:*K*^,

(6) $$P\left(\Theta |T^{1:K}\right)=\frac{P\left(T^{1:K}|\Theta \right)P(\Theta )}{P\left(T^{1:K}\right)}=\frac{\overset{K}{\underset{k=1}{\prod }}P\left(T^{k}|\Theta \right)}{P\left(T^{1:K}\right)}P(\Theta ),$$

where *P* (Θ) = *P*(*θ*) · *P*(*B*) · *P*(*κ*) is a jointly independent parametric prior and *P*(**T**^**1**:**K**^) is the marginal over the decision time set, which can be dropped as it does not depend conditionally on model parameters. The MAP estimate for model parameters $\hat{\Theta }$ is then the mode of the posterior, Eq. (6),

$$\hat{\Theta }={\text{argmax}}_{\Theta }P\left(\Theta |T^{K}\right)={\text{argmax}}_{\Theta }\overset{K}{\underset{k=1}{\prod }}P\left(T^{k}|\Theta \right)P(\Theta ),$$

selecting (*θ*, *κ*, *B*) for diffusive and pulsatile coupling and (*θ*, *B*) for no coupling.

**FIG. 5.**
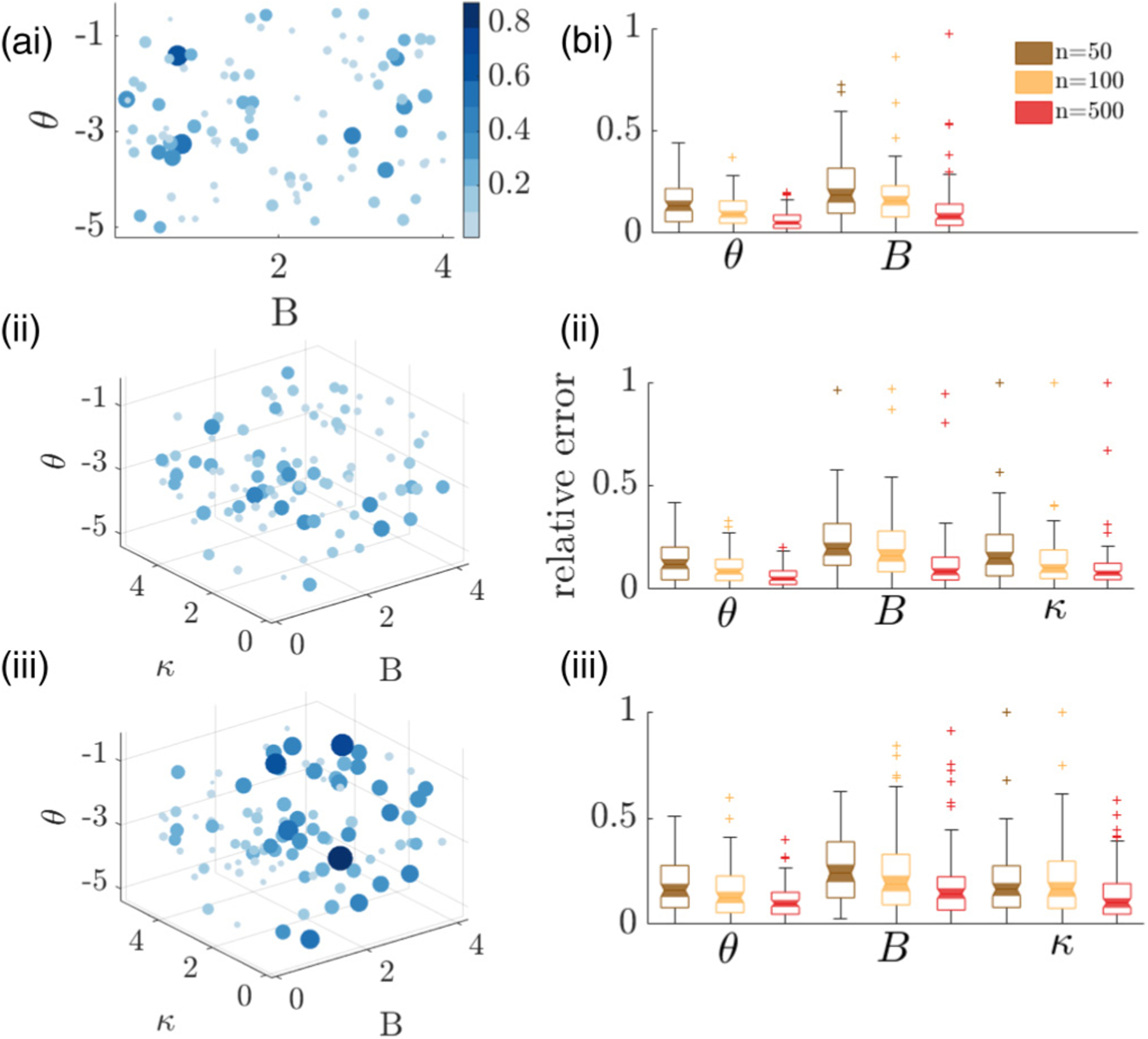
Parametric fits of no coupling, diffusive, and pulsatile model using Bayesian maximum a posteriori (MAP) estimation. (a) Estimation errors for (i) no information coupling, (ii) diffusive coupling, and (iii) pulsatile coupling. Bubble location represents true parameters and bubble size/color indicates the total sum of relative estimation error for displayed parameters, Eq. (7). Each plot is obtained from 100 parametric samples from a uniform prior where *θ* ∈ [−0.1, −5], *κ* ∈ [0.1, 6], *B* ∈ [0.1, 4] given *n* = 100 departure time samples each. (b) Box plots showing the median relative error (notches), upper/lower quartiles (boxes), minimum/maximum excluding the outliers (error bars), and outliers (stars) in MAP estimate of the model parameters (*θ*, *B*, *κ*) for (i) no information coupling, (ii) diffusive coupling, and (iii) pulsatile coupling models, calculated using data of sample size *n* = 50, 100, 500. The displayed distributions of the relative error were computed using 100 parametric samples from a uniform prior.

We fit models to data generated by a model whose models are randomly chosen from the parametric prior [Fig. 5(a)], and compute the error relative to the true parameters used to generate synthetic data. For the coupled models,

(7) $$\text{Rel Err}=\frac{1}{3}\left(\frac{\left|\hat{\theta }-\theta^{\text{true}}\right|}{\left|\theta^{\text{true}}\right|}+\frac{\left|\hat{B}-B^{\text{true}}\right|}{B^{\text{true}}}+\frac{\left|\hat{\kappa }-\kappa^{\text{true}}\right|}{\kappa^{\text{true}}}\right),$$

and an analogous expression is used for the model with no coupling. Parameters for the pulsatile coupled model have the highest relative error, but we find no other systematic variation in error. We conjecture that the higher relative error of the pulsatile coupled model is due to its parameter sensitivity. As such, sampling variability in generating synthetic data makes parameters more difficult to identify.

We also compare the error in parameter estimation for different sample sizes of departure times for the three different models across the parametric prior [Fig. 5(b)]. The box plot shows the relative error in the MAP estimate of model parameters for data of sample size *n* = 50, 100, and 500. The average error is slightly higher for the pulsatile model, consistent with the results for individual parameter sets in Fig. 5(a). Generally, increasing the number of decision time pairs sampled tends to decrease the average relative error. One exception is the coupling parameter *κ* in the pulsatile coupling model [Fig. 5(b)(iii)], which we expect is due to stochasticity in the calculation of the effective average relative error.

**FIG. 6.**
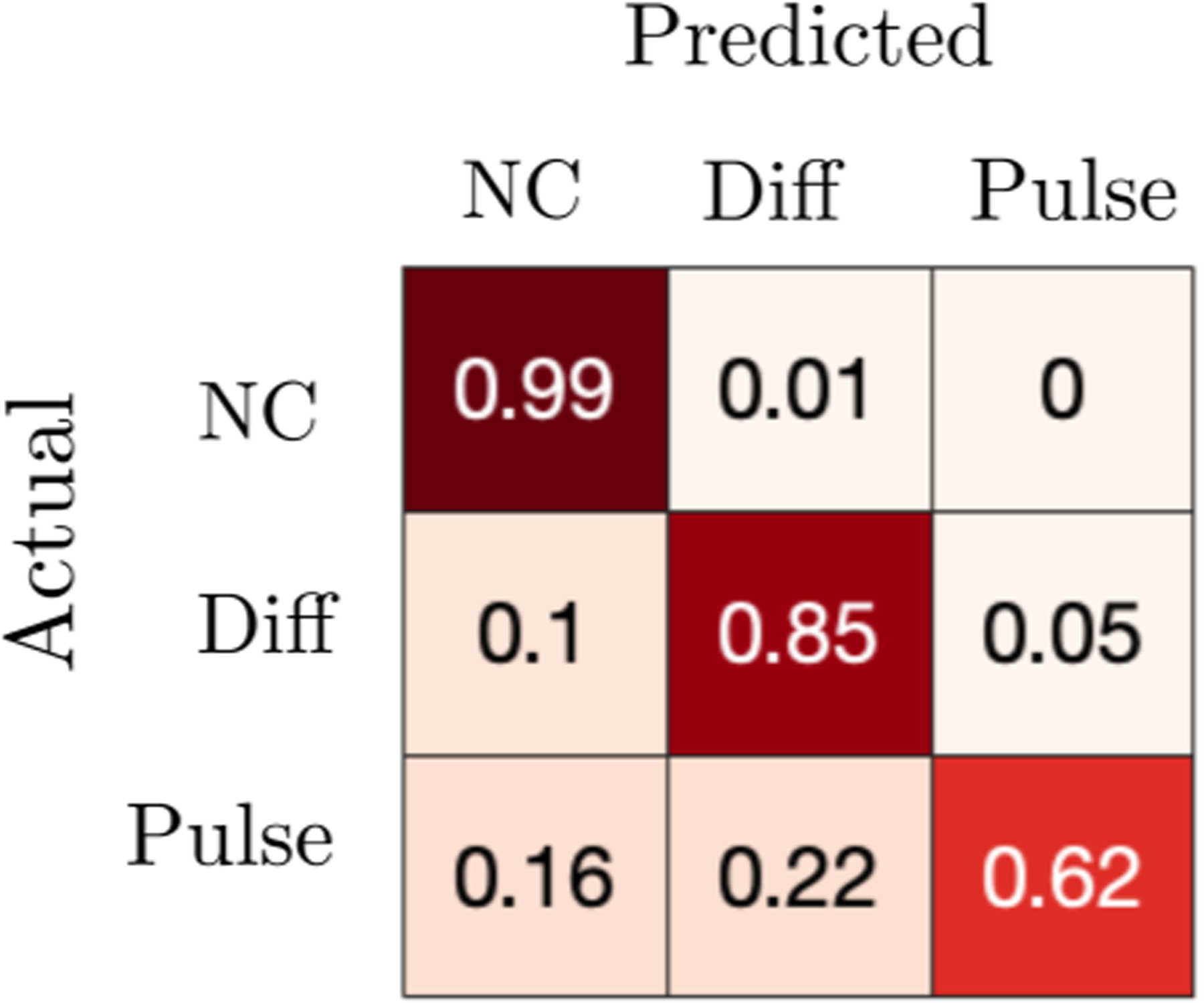
Confusion matrix showing model identifiability for no coupling (NC); diffusive coupling (Diff); and pulsatile coupling (Pulse). Numbers and heatmap represent fraction of actual model samples predicted to be each model type, based on a Bayes Factor analysis, Eq. (8). Each row is generated from 900 parametric samples of the actual model using a uniform prior on *θ* ∈ [−5, −0.1], *κ* ∈ [0.1, 6], *B* ∈ [0.1, 4]. Each model fit uses *n* = 50 decision time pairs.

**FIG. 7.**
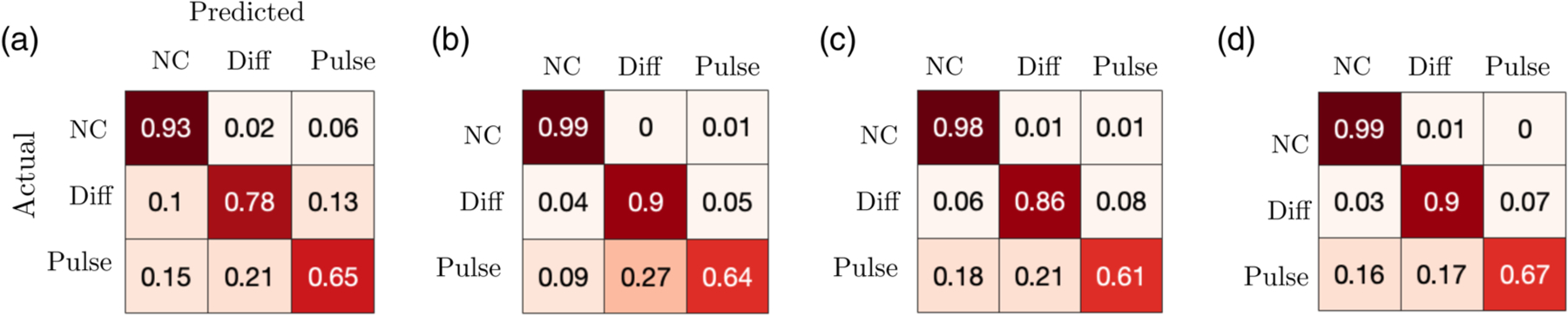
Confusion matrix showing identifiability of model classes using data of sample sizes (a) *n* = 10; (b) *n* = 100; (c) *n* = 500; (d) *n* = 1000. Model parameters are drawn randomly with uniform prior as in Fig. 6.

To develop a framework for determining the modes of communication used by foraging animals, we calculate Bayes factors to determine which of two model classes of a pair better explains data. Bayes factors are a model comparison measure defined as the ratios of the likelihood of either model class across all possible parameter values [65]. Considering two such models {*M*_1_, *M*_2_} ∈ {*M*_no coupling_, *M*_diff_, *M*_pulse_}, we can define the log likelihood ratio of either model *M*_*j*_ given a set of observed decision times and then use Bayes rule to rearrange

$$\text{log BF}=\text{log}\frac{P\left(M_{1}|T^{1:K}\right)}{P\left(M_{2}|T^{1:K}\right)}=\text{log}\frac{P\left(T^{1:K}|M_{1}\right)P\left(M_{1}\right)}{P\left(T^{1:K}|M_{2}\right)P\left(M_{2}\right)},$$

where **T**^1:*K*^ is the set of *K* patch departure decision time pairs. For equally likely models *P*(*M*_1_) = *P*(*M*_2_), and we can marginalize over parametric priors to obtain

(8) $$\text{log BF}=\text{log}\frac{\int_{\Omega_{\Theta_{M_{1}}}}P\left(T^{K}|\Theta_{M_{1}}\right)P\left(\Theta_{M_{1}}\right)d\Theta_{M_{1}}}{\int_{\Omega_{\Theta_{M_{2}}}}P\left(T^{K}|\Theta_{M_{2}}\right)P\left(\Theta_{M_{2}}\right)d\Theta_{M_{2}}},$$

where $\Theta_{M_{j}}$ is the set of parameters for model *M*_*j*_ with corresponding parametric prior $P\left(\Theta_{M_{j}}\right)$ defined on the set $\Omega_{\Theta_{M_{j}}}$. Positive (negative) log Bayes factors provide evidence for model *M*_1_ (*M*_2_). For a randomly chosen parametric set of an actual model, the predicted model is the one that always generates logBF > 0 when in the numerator (*M*_1_). To determine how difficult it is to identify each model, given 900 parametric samples from a uniform prior for each model, we empirically generate a likelihood of each model class being identified as itself or another model (Fig. 6). The model without coupling is easily identified whereas the coupled models are misclassified more often. For instance, using a coupled model with weak coupling parameters can lead to misidentification as a model with no coupling. The pulsatile coupled model is the most difficult to identify. We conjecture this is because of its goodness-of-fit sensitivity to the coupling parameters (which is penalized by Bayes factors). The diffusive model, on the other hand, is a more robust model, so it is identified more often, and also often predicted when synthetic data from the pulsatile model is provided. Note, these results do not change significantly as the number of decision time samples is increased (see Fig. 7).

We conclude that the pulsatile model can perform well despite only generating one moment of information exchange per trial, but does require fine-tuning and can be difficult to identify. The diffusive coupled model is more robust and does require fine tuning, but involves continuous information exchange. Our model identification results suggest that we can expect reasonable model class identifiability even with only *n* = 50 patch departure decision observations for a pair, suggesting our analysis could translate well to identifying the strategies used by animals in the field.

## IV. DISCUSSION

Many animals forage in groups, but most models greatly simplify the decision formation and belief sharing processes of groups or only focus on the mechanics of single foragers patch departure decision process. In this paper we extended our previous mechanistic models of patch foraging decisions [17,18] to consider social information sharing among a cohesive foraging group. Group cohesion is formulated as a constraint, and represents the movement dynamics of animals, for example, baboons or capuchin monkeys, which tend to move together. Individuals in the group must not only infer the current state of resources to choose an efficient time to leave the patch, but also must synchronize decisions for the group to stay together. Poor synchronization decreases the group reward rate, due to wasted time of early deciders. Information sharing not only helps synchronize the group but also improves the accuracy of noisy or imperfect decision processes.

We considered two different form of information coupling—diffusive (full sharing of an agent’s belief about the current patch quality) and pulsatile (sharing only of time of decision to leave). We asked how these couplings affect overall foraging efficiency, and further examined how the coupling parameters and type can be inferred from data. Strong coupling leads to efficient group decisions (i.e., maximizing the average reward among group members) through synchronization of departure decisions. Over the parameter ranges we tested, pulsatile coupling can yield similar or higher average return rates than diffusive coupling because it more readily promotes group synchronization in decision times. On the other hand, the pulsatile model must be fine tuned, since it generates singular communication events, so performance falls off more rapidly than in the diffusive coupled model.

We determined the identifiability of model parameters from data generated by the models themselves. Both coupling and decision threshold parameters were accurately inferred when the model class was known. Although, the pulsatile model’s parameters were more difficult to identify, likely due to the model’s sensitivity. When the model class was not known, the diffusive coupled model was occasionally misclassified (mostly as a model with no coupling) and the pulsatile model was misclassified more often (typically as a diffusive coupled model). Model parameter and class identification performance was limited to that obtained using 10 to 100 decision time pairs, a feasible volume of social foraging field data [66–68]. Our approach provides a clear framework for identifying information exchange strategies during social patch foraging, and for fitting models to data.

Other factors still can complicate the decision of whether or not to depart a patch, beyond an agent’s estimate of available resources. Classic theory has considered the marginal value theorem (MVT), based on the principle that animals will tend to depart when resource availability wanes below a density at which current patch exploitation would be more profitable than departing and exploiting another patch [69]. However, this approach neglects the variability extant in animals’ resource density estimates, which can strongly shape their patch departure statistics [17,18]. Threat of predation is another factor that might affect an animal’s decision to leave a patch [70]. Remaining in the same location for too long puts one at risk, so animals may leave well before predicted by the MVT [71]. Such threats can even be paired with that of competition from species foraging similar resources, so as to further complicate the decision to depart a patch [72]. Vigilance in response to possible threats can also shape the ongoing rate of resource consumption, as animals must heed the potential for threats as they feed [73]. Such factors could be accounted for in our model by considering predator threat costs in the reward rate function or drifts that would balance considerations of resource attainment with animals’ vulnerability during foraging bouts.

Some efforts have been made to identify group-wide principles of the MVT for those that leverage social information. A previous study suggested members of groups should leave sooner than if they were alone, each gaining less energy than single animals exploiting the same patch [74]. Predictions of patch departure statistics were validated using spice finches, exploiting food patches alone or in groups of three under two habitats that required different travel times. Group members left the patch sooner and with fewer seeds than solitary foragers, but birds did not share the patch equally and their patterns of exploitation could not be predicted by single forager models. Moreover, within each group, the bird expected to leave first delayed its departure although it collected fewer seeds than the others, perhaps attempting to maintain cohesion. Birds seemed to depart at the same time, as a unit, and there was no preferential order of departure measured across patches. Birds seemed to assess patch richness by observing their conspecifics’ feeding rates, combining this “public information” with personal information derived from their own foraging success [21,75]. Despite differences in individual feeding rates, all group members can leverage social information to obtain the same estimate of patch richness, suggesting they should depart simultaneously [76]. Among other bird species, starlings appear to use public information during foraging [75], while budgerigars do not [76].

Indeed there are a number of possible extensions to the model we could consider, relevant to identifying collective decision strategies of foraging groups and tractable models that might admit explicit departure time expressions. Note, we could incorporate time-varying noise into the drift-diffusion process to better represent the nonmonotonic changes in food encounter rate variance as the forager depletes the patch. Such an assumption could result in tighter patch departure distribution peaks as the variance would wane as the patch is depleted. Also, some animals do forage in large (*N* ≫ 1) groups [77], so that (a) diffusive coupling could significantly dampen noise in the patch quality estimate or (b) pulsatile coupling could lead to rapid departure of all animals in a connected group [78]. Model statistics in such large system-size limits could be obtained using mean field approximations or moment closure approximations, which would account for the dominant motifs in the social structure network [79]. To account for the energy involved in communication, we could consider a cost in the reward rate function either scaling linearly with time in patch for diffusive coupling or a smaller one-time cost for pulsatile coupling.

Variations in social hierarchy and energy seeking aims can also arise within animal groups. First, while some animal groups move as a cohesive whole on the landscape, other groups (for example, spider monkeys) have fission-fusion dynamics. One can relax the assumption that foragers leave together, and represent fission-fusion group dynamics by considering agents that move between patches in a foraging landscape. Second, one can introduce a variety of biases that animals exhibit into these models such as satisficing or state-dependence (e.g., hunger or thirst) [80]. Third, one can study effects of social structure observed in different animal groups, such as either hierarchical or egalitarian [81], as well as forms of coupling on the foraging dynamics, opening up the opportunity to link collective social structure to collective and individual foraging dynamics.

One category of previous foraging models focuses on the problem of two-dimensional stochastic movement dynamics and efficient modes of spatial exploration and search [82]. Our model simplifies this interesting and complementary aspect of the foraging problem, since we wished to focus on how social influence shapes the cognitive processes underlying the decision to leave a local resource region and search for another. One commonly modeled and experimentally identified search strategy involves local diffusive search interspersed with longrange ballistic motion [83]. Such intermittent search strategies are consistent with the framework of patch foraging, which also involves local exploitation of patches punctuated by interpatch travel [84]. Statistics of these strategies have often been characterized by power law (Lévy flights) or gamma transit length distributions [85,86]. In future work, we could consider models that also identify how social interactions can steer transits between patches or local foraging bouts to see how this shapes the efficiency of search strategies. It would also be interesting to consider foraging strategies of agents whose environments are not strictly patchy but are sampled locally due to finite spatiotemporal precision [87]. In this case, the decision to leave a local region may still follow the deliberation and commitment process laid down here for patch foraging.

In previous paper, we considered Bayesian agents that could learn the resource distribution in an environment as they visit multiple patches [88]. Such a model calls into question the primary goal of maximizing reward rate, since rapid learning may require overharvesting patches. Aside from needing to learn their environmental context, there are a number of factors (predation, season, reproductive state, or competition) that could affect patch departure decisions that could evolve dynamically over time [84].

Social foraging is crucial for animals as it is important for resource localization and collective search [15,66,89–93]. While we considered cohesive group movement, other animals such as spider monkeys live in groups but do not maintain cohesion during daily foraging, instead leaving and rejoining groups as they forage (so-called fission-fusion group dynamics) [94,95]. An extension of our modeling approach could be used to represent fission-fusion group dynamics by considering agents that move between patches in a foraging landscape. Factors other than reward optimization also play a strong role in driving group organization including predator protection, environmental constraints, and mating behavior. Current work seeks to examine evolutionary drivers of differences in group social dynamics within and across species, which could provide a broader class of group performance measures and communication modalities for quantitative models [16,68].

Our model represents patch-leaving decisions using an accumulation-to-bound process [17], where individuals incorporate both personal and social information in order to determine when to leave a patch. Linear diffusive coupling has been shown to generally reduce noise in belief estimates [44], but it is important to consider various forms of information sharing (e.g., diffusive versus pulsatile coupling) and the way in which such shared information is translated into a decision. Other work has considered nonlinear (e.g., sigmoidal) interactions and information sharing in social groups [96,97]. For larger groups, an alternative representation could treat beliefs as a “complex contagion,” based on a fraction of connected neighbors that have made a decision, instead of a simple sum [41,98]. Furthermore, individuals may not be uniformly connected to others so that influential individuals have more weight in their information sharing with neighbors [81,99,100], is an interesting further extension of our model approach, and can help inform how different species exchange information [101].

To socially forage, animals often use social cues, informing their decisions through a variety of sensory modalities: the observed harvest or departure of conspecifics from patches [102], olfactory cues (e.g., informative breath [103]); vocalizations [104–106]; or visual signals [107]. Birds and other species have broad-ranging sensory abilities to detect conspecifics: scavengers can visually detect a conspecific circling a carcass from many kilometers [67], and marine birds can spot diving neighbors [108]. Our model forms a basis for future studies that could incorporate more nuanced spatiotemporal features and modalities of communication.

Increasingly sophisticated recording technology facilitates high-resolution motion tracking of diverse species [109,110], allowing for a thorough validation of theoretical models [111]. The availability of such technologies and the gathered large scale data can allow model fitting to foraging behavior; e.g., building on the use of drift-diffusion models fit to evidence accumulation or visual search tasks [112]. By fitting to simulated data, we showed that the parameters in our model can be readily identified, but if the coupling type is unknown, inferring the pulsatile coupling strategy and its parameters is more difficult than inferring diffusive coupling. Future work can build on these results to fit to social foraging data, and infer the information-sharing strategies and differences among individuals.

Software generating model statistics, identifying models, and plotting figures can be found at GitHub [113].

## APPENDIX

### 1. Moments of resource depletion model

Consider the depletion process by which a forager encounters food chunks in a patch at a rate *λ* · *a* proportional to the current number *a* of food chunks left, immediately consumes a chunk when encountering, and continues until leaving the patch or consuming all food chunks. In this case the number of chunks left evolves stochastically as a pure death process, and the probability of there being *a* chunks left at time *t* is given by the coupled system $p_{A}^{\prime }(t)=-\lambda Ap_{A}(t);p_{a}^{\prime }(t)=\lambda (a+\text{ 1) }p_{a+1}(t)-\lambda ap_{a}(t)\;(a=1,\ldots ,A-1);\;p_{0}^{\prime }(t)=\lambda p_{1}(t)$. Assume initially there are *A* chunks of food [*p*_*A*_(0) = 1]. To determine the first moment (mean) for the number of chunks left at time *t*, we define $\mu_{1}(t)=\sum_{a=0}^{A}ap_{a}(t)$ so that $\mu_{1}^{\prime }(t)=\sum_{a=0}^{A}ap_{a}^{\prime }(t)$. As such, we can define the differential equation

$$\mu_{1}^{\prime }(t)=-\lambda \overset{A}{\underset{a=1}{\sum }}a^{2}p_{a}(t)+\lambda \overset{A-1}{\underset{a=0}{\sum }}(a+1)ap_{a+1}(t)\;=\lambda \overset{A}{\underset{a=1}{\sum }}\left[a(a-1)-a^{2}\right]p_{a}(t)=-\lambda \mu_{1}(t),$$

with initial condition *μ*_1_(0) = *A*, so *μ*_1_(*t*) = *A*e^−*λt*^. Since the encounter rate increases linearly with the number of foragers, if there are *N* foragers, we would analogously find *μ*_1_(*t*) = *A*e^−*λNt*^, so *N* can be absorbed into the rate *λ*. Considering now the second moment $\mu_{2}(t)=\sum_{a=0}^{A}a^{2}p_{a}(t)$, we can similarly formulate an evolution equation

$$\mu_{2}^{\prime }(t)=-2\lambda \mu_{2}(t)+\lambda \mu_{1}(t)=-2\lambda \mu_{2}(t)+\lambda A{\text{e}}^{-\lambda t}$$

with initial condition *μ*_2_(0) = *A*^2^, which can be solved to find *μ*_2_(*t*) = (*A*^2^ − *A*)e^−2*λt*^ + *A*e^−*λt*^. Variance is then easily computed as *σ*^2^ = (*A*^2^ − *A*)e^−2*λt*^ + *A*e^−*λt*^ − *A*^2^e^−2*λt*^ = *A*e^−*λt*^ (1 − e^−*λt*^), which rises and falls as resources are consumed. As with the first moment (mean), instantaneously changing the number of foragers will instantaneously change the rate *λ* as a piecewise smooth process. As such, we can compute the mean piecewise in time, adapting the rate for each forager count.

### 2. Departure time statistics calculation

Decision and departure time statistics are obtained either by running Monte Carlo simulations (on the order of 10^5^ per parameter set) or simulating the corresponding Fokker-Planck equation and calculating the flux through decision boundaries at this *x*_1_ = *θ*_1_ and *x*_2_ = *θ*_2_. Although the model formulation is general, we focus the analysis in this paper on the tractable case of two coupled agents.

#### a. Monte Carlo simulations

One straightforward way to approximate departure time statistics is to perform many realizations of the stochastic differential equations describing agents’ beliefs. For a pair of agents, a single Monte Carlo simulation initializes (*x*_1_(0), *x*_2_(0)) = (0, 0) and employs the Euler-Maruyama algorithm with the time step Δ*t* = 0.01 for the corresponding pair of coupled Langevin equations until either *x*_1_(*t*) ⩽ *θ*_1_ or *x*_2_(*t*) ⩽ *θ*_2_. Thereafter, for diffusive coupling the remaining agent’s belief *x*_*j*_ evolves with *x*_*k*_ replaced by *θ*_*k*_ until *x*_*j*_ ⩽ *θ*_*j*_. For pulsatile coupling, the remaining agent’s belief is instantaneously incremented *x*_*j*_(*t*) − *κ*_*j*_ and evolves until *x*_*j*_ ⩽ *θ*_*j*_. The resulting first and second decision times *T*_1_ and *T*_2_ are recorded and used to compute statistics.

#### b. Fokker-Planck equation simulations

When the group’s decision time can be described by a single variable system (perfectly coupled cases considered in Sec. IIIA), the departure time statistics are computed by numerically simulating the associated Fokker-Planck equations. The Fokker-Planck equation takes the form

(A1)
$$\frac{\partial P(x,t)}{\partial t}=-\frac{\partial }{\partial x}\left(\left(\rho e^{-\frac{Nt}{\tau }}-\alpha \right)P(x,t)\right)+\tilde{B}\frac{\partial^{2}}{\partial x^{2}}P(x,t),\;P(\theta ,t)=0,\;\left(\rho e^{-\frac{Nt}{\tau }}-\alpha \right)P(L,t)=\tilde{B}\frac{\partial }{\partial x}P(L,t),$$

and is defined over the domain [*θ*, *L*], where *x* = *θ* is an absorbing boundary and *x* = *L* (*L* sufficiently large) is a reflecting boundary. The value of $\tilde{B}$ is *B*(*B*/2) for pulsatile (diffusive) coupling. Eq. (A1) is numerically simulated using a second order finite difference method in both time and space dimensions with Δ*t* = 0.005 and Δ*x* = 0.1 to obtain the probability density for the decision variable *x*, *P*(*x*, *t*). The time dependent probability of departure decisions is obtained by calculating flux through the absorbing boundary *x* = *θ*.

### 3. Likelihood functions calculation

Likelihood functions *P*(**T**|Θ) used for model identification and model fitting were obtained by solving the associated Langevin equations using Monte Carlo simulations for a discrete set of parameters, which covers the space of the uniform prior. We discretized the parameter space Θ into a three-dimensional grid (*θ*, *B*, *κ*) ∈ [−5, −0.1] × [0.1, 4] × [0.1, 6] with 40 partitions along each dimension. Decision time distributions for each parameter set are binned as a probability mass function along (*T*_1_, *T*_2_) ∈ [0, 75] × [*T*_1_, 75] with steps of size Δ*T* = 0.5 along each dimension.

### 4. Model identification with data samples of varying length

Here show the general trends reported above (Fig. 6) are preserved when identifying model class using various volumes of decision time sets (Fig. 7). The model without coupling is easily identified whereas the coupled models are more likely to be misidentified, especially the pulsatile coupled model. Moreover, note there is not substantial improvement in the model class identification fractions when increasing to *n* = 1000 samples per model fit.
